# Supplementary material for: Sonic Hedgehog Is a Member of the Hh/DD-Peptidase Family That Spans the Eukaryotic and Bacterial Domains of Life
Source: J Dev Biol. 2018 Jun 8;6(2):12. doi: 10.3390/jdb6020012 (PMC6027127; doi:10.3390/jdb6020012)
Supplement: Supplementary file 1 [file jdb-06-00012-s001.pdf]

Appendix A

Accession numbers for the proteins used for the lineup in Figure 3.

WP_082159544.1	Hypothetical protein [Micromonospora sp. HK10]	
WP_098188151.1	Hypothetical protein [Bacillus pseudomycoides]	Related Information
WP_028591995.1	Hypothetical protein [Paenibacillus panacisoli]	
WP_069829738.1	Hypothetical protein [Algibacter aquaticus]	Related Information
WP_080055297.1	Hypothetical protein [Spirosoma aerolatum]	
WP_103381118.1	Hypothetical protein [Pseudonocardia dioxanivorans]	
ESZ39647.1	Hypothetical protein X732_15330 [Mesorhizobium sp. L2C066B000]	
WP_029077494.1	Hypothetical protein [Bradyrhizobium sp. th.b2]	
WP_040135141.1	Hypothetical protein [Pseudoalteromonas piratica]	Related Information
ABX84114.1	Hedgling [Nematostella vectensis]	
XP_022667503.1	Indian Hedgehog protein-like [Varroa destructor]	Related Information
ABX89897.1	Hedgehog1 [Nematostella vectensis]	Related Information
ABX89898.1	Hedgehog2 [Nematostella vectensis]	Related Information
NP_001034065.1	Hedgehog [Drosophila melanogaster]	Related Information
XP_007124731.1	Sonic Hedgehog protein [Physeter catodon]	

Appendix B

BLAST Multiple alignment input (ncbi.nlm.nih.gov) for Hh/DD-peptidase domains used for the lineup in Figure 4

>Algibacter

LKLKERVPNMKEYDVVGRISGKILRGTPEFDELIKNENQKIVFKDEEGTGADHYMTSKLSEKLNLL
ADLVIQEWGANIKLRVTEAWDEDNEHASKSIHYEGRGADITTSDRDSSKLGRLARLAVEVGLDW
VFYEDNSHVHVSMKK

>Bacillus

LNLGEKVPNKKESDVVGPITGVIQRGNPDFNTLVRSNNQDIQFKDEEGTGADFLMTSRLSDKLNT
LAILVNQEWPNIKLRVTEAWDEDNEHSSGSTHYEGRAADITTSDRDGNKLGRLAQLAVDAGFD
WVYYENKYHIHVSVKK

>Bradyrhizobium

LTQGQKVPNASETATCGAIAKKIKRTDPEFATLVSNQNASIVFKDEEGTGADRMMSTRLQAKLD
ALASLVSAEWAGVKLRVTEAWDENDEHLPTALHYEGRAADITTQPPDGAKLGRLARLAVNAG
CDWVFYEDTSHVHVSVKKA

>Pseudonocardia dioxanivorans

VPLGEH
VPARAEADASGAATGVVAAGSPEFDALVRLDDPTVVVKDEEGSGADRMMTPRLAELVGVLAA
HVAQAFPGRRLRLTEAWDPDGEHSHSSLHYEGRAADLTVDDRDRAKLGRLAALAVQTGFDWV
LHENDHVHVSVRAG

>Mesorhizobium

LSQGQKVPNASELVTCGPITKKITRTDPEFAGLVTNTNAKIVFKDEENTGADRMMTPRLKSKLDS
LANVVASEWPGAKLRVTEAWDEDNEHADASLHYEGRAADLTTNPVDGAKLGRLARLAVDAG
CDWVFFEDSSHIHVSVKAG

>Micromonospora

LSLGERVPNVGEASVVGPIAGKVVRGSPEFNALVKNDNPDIVFKDEEKTDADRMMTPRLRDMV
NELAALVVKEWPGKKLRVTEGWDENNEHTAESTHYEGRAVDMTVSDLDAAKLGRLARLAVD
AGFDWVFYENALHVHASVKK

>Nematostella Hh1

LYFKQRVPDVDEFSLGASGRPQGKITRNSSKFNKLVACYNTDIVFKDEERTGADRLMSKRCREKL
RNLATKVKQKWKGVKLRVTEAWDEDGQHSLDSLHYEGRAVDISTSDKDPKKLPDLGSLAVDAG
FDWVYYDRRSSIHASVRS

>Nematostella Hh2

MKIREHIPDTSETSLQASGPSRKIKRGSNGYKELITNADPNIVFREDKAGNNRRMSKRCERKLKILS
SLVRKEWIGDVKVRVIRAYDDGTSKKRHHGPHSLHFSGRALDITTSDEKRDKLPMLGRLAYRAGF
DWVYRAKAYIHASVKS

>Nematostella vectensis hedgling

LSQGSSYPYSKTEVSVCGGIRDVILRNSARFRKILVRNADTEVVFENDDCRRTTARAKSKLDVLAS
RVRQEWAGRKLKVIKAWTDQRTAQDPASLHYEGRALRLQLDNNDRSMLSRLAGLALASGFDW
VSYPLNSDYIHASVIR

>Paenibacillus panacisoli

LSVGQKVPDMLESQAAGPFTDAVHRGSNEFKNFVYNNNPLIVYDDEERGHSNHYMTKDLADQ
LNRLAELVAAEWSGIKLMVIDGWSDPDVRPPHNNRLYHEGRAADLTVSDLDTSKLGRLGWLAV
DAGFDFVHYESQDHIHVAVKV

>Pseudoalteromonas piratica

LKKGQRWPQSSEIKSSGEARQTLSGSSLRDCMNKSANNLPVFKFEEGNFTDVQASEKLCAAIMDL
NKLVMKEWPGKTLRVTEAYDQDGEHAKFSLHNEGRAADMTVSDRDLKKLGRLGFLATKAGFS
WVYYEHNHIHASVKR

>spirosoma

LTLGQKVPDRAEKDSLASGPFNKAILRGTPEFATLVENKNEKVVFKNEEGDGSDRMMTPVLKTH
VDRLADLVRSEWGAGVSLRVTEAWDDTGEHSSSHSLHYEGRAVDLTTSDLDKSKLGRLGRLAVD
AGFNWVYYENLLHIHASVTKA

>Varroa destructor Hh

LVVKQYVPNTAEQNEIAAGRAKGAISRTSMAFRKLVPNYNTDIRFLDDEGTGADRIMTQRCRDK
LDTLAVSVMTQWPGVKLRVIESWDEYSHHKSGSLHYEGRAVDFTTDDRHQAKYGMLARLAVE
AGFDWVYYETKRHVHASVKP

>Drosophila Hh

KQTIPNLSEYTNSASGPLEGVIRRDSPKFKDLVPNYNRDILFRDEEGTGADRLMSKRCKEKLNVLA
YSVMNEWPGIRLLVTESWDEDYHHGQESLHYEGRAVTIATSDRDQSKYGMLARLAVEAGFDWV
SYVSRRHIYCSVKS

>Sperm Whale Shh

LAYKQFIPNVAEKTLGASGRYEGKITRNSERFKELTPNYNPDIIFKDEENTGADRLMTQRCKDKL
NALAISVMNQWPGVKLRVTEGWDEDGHHSEESLHYEGRAVDITTSDRDRSKYGMLARLAVEAG
FDWVYYESKAHIHCSVKA
